# Unqualified Practice at Hospitals

**Published:** 1894-11-24

**Authors:** 


### Unqualified Practice at Hospitals.

A correspondence in the *Times* has drawn attention to the extent to which unqualified students are entrusted with the actual care and treatment of patients at some of our London hospitals. It is a question which is continually cropping up, and one upon either side of which it is easy to say a good deal. Educationally there can be but little doubt that it is a great advantage to senior dressers, who, except that they have not actually passed their examinations, are as fully read as many qualified men, that they should have an opportunity of taking that actual charge of patients which will be their work in after life, and of doing so while help is at hand, *i.e.*, under circumstances which give the greatest security to the patients; nor can we shut our eyes to the fact that, organised as so many of our hospitals are, with their enormous crowds of out-patients, the employment of dressers in the treatment is a great administrative facility, for it it is an absolute necessity that there should be a large staff at hand to get through the mass of work which must be crowded into a comparatively short space of time, unless patients are to waste even more time than they do at present waiting to be seen; it may, moreover be granted without risk of denial, that unless our system of medical education is an utter and absolute failure, men in the position of senior dressers ought to be fully competent to treat the comparatively trivial diseases from which so large a proportion of the applicants at our hospitals seem to suffer. On the other hand, one must not forget that the hospital authorities, in handing over the treatment of patients to unqualified men, are sanctioning a proceeding which the full force of public opinion as expressed, and to some extent enforced, by coroners, magistrates, judges, juries, and the press, has for long been trying to put down. A general practitioner who hands his patients over into the entire charge of an unqualified assistant cannot recover his fees for such attendance, and the death of a patient under such treatment will in all probability be brought before the coroner.

A doctor who has to be away from home visiting his patients half the day may employ an unqualified assistant to attend to the surgery and dispense his medicines; but woe betide him if this unqualified man runs across the street to attend to an accident, or rushes off with the stomach pump in response to an urgent summons to a case of poisoning. If the case goes wrong, ten to one the doctor gets a wigging from the jury for employing an unqualified assistant, and if it should turn out that he pays this assistant a percentage as well as a salary, he may think himself lucky if he escapes pains and penalties from the Medical Council for the professional crime of "covering." Yet it is asserted that at hospitals, under the cloak of charity, all these things can be done.

Of course it is said that the dressers merely look through the great mass of applicants, treating only the trivial ailments, and that all the cases of any gravity are passed on to the surgeons. To this the retort is obvious, that the decision as to what is trivial and what is not really requires more skill than the mere treatment of a disease the name and gravity of which is known. After all the question is one of honesty. So long as the law maintains a distinction between qualified and unqualified men, so long as hospitals remain the exponents of qualified practice, and so long as the names of a qualified staff are made known to the public, as those of the men by whom the patients are treated, so long it cannot be right that patients applying at these hospitals should be handed over to the treatment of men who possess no legal qualification to practise. Nor do we think any real difficulty or hardship need arise from putting in force the plain dictates of honesty in this matter. It is not a question as to whether students shall be allowed to see patients, or even to treat them; it is entirely one of exclusive charge. The aim should be to put the hospital on the same footing as the private practitioner in this matter. The assistant may assist, but he may not take entire charge; so, surely, the student may assist, may carry out a line of treatment, may watch a case and report, may even undertake certain operative measures under the eye and direction of the master; all these things are good both for the student and the patient, and they are honest. It ought, however, to be understood that when a patient goes to a hospital he will be certain to have his case investigated by qualified men and treated at the least under their direction.

